# Phosphorylation of Cdc5 regulates its accumulation

**DOI:** 10.1186/1747-1028-6-23

**Published:** 2011-12-28

**Authors:** Kobi J Simpson-Lavy, Michael Brandeis

**Affiliations:** 1 The Department of Genetics, The Silberman Institute of Life Sciences, The Hebrew University of Jerusalem, Jerusalem 91904, Israel; 2 Dept. of Biochemistry and Molecular Genetics, University of Colorado-Denver, 12801 E. 17th Ave, Aurora CO 80045, USA

**Keywords:** Cdk1, Cdc28, Clb2, Swe1, Polo, mitosis, phosphorylation, APC/C, Cdh1

## Abstract

**Background:**

Cdc5 (polo kinase/Plk1) is a highly conserved key regulator of the *S. cerevisiae *cell cycle from S-phase until cytokinesis. However, much of the regulatory mechanisms that govern Cdc5 remain to be determined. Cdc5 is phosphorylated on up to 10 sites during mitosis. In this study, we investigated the function of phosphorylation site T23, the only full consensus Cdk1 (Cdc28) phosphorylation site present.

**Findings:**

*Cdc5^T23A ^*introduces a degron that reduces its cellular amount to undetectable levels, which are nevertheless sufficient for normal cell proliferation. The degron acts *in cis *and is reversed by N-terminal GFP-tagging. Cdk1 kinase activity is required to maintain Cdc5 levels during G2. This, Cdk1 inhibited, Cdc5 degradation is APC/C^Cdh1 ^independent and requires new protein synthesis. Cdc5^T23E ^is hyperactive, and reduces the levels of Cdc5 (*in trans*) and drastically reduces Clb2 levels.

**Conclusions:**

Phosphorylation of Cdc5 by Cdk1 is required to maintain Cdc5 levels during G2. However, phosphorylation of T23 (probably by Cdk1) caps Cdc5 and other *CLB2 *cluster protein accumulation, preventing potential protein toxicity, which may arise from their overexpression or from APC/C^Cdh1 ^inactivation.

## Findings

Cdc5/polo kinase is a crucial player in cell-cycle regulation from yeast to man, and the processes and substrates it regulates have been extensively investigated. Cdc5 regulates numerous cell cycle events, including promoting the destruction of the Cdk1-Clb2 inhibitor Swe1 [1], *CLB2 *cluster transcription (including itself) [2], spindle-pole body separation [3], spindle positioning [4], microtubule organization [5], recovery from hydroxyurea [6], APC/C activation [7], mitotic exit [8], cytokinesis [9,10], Cdc14 localization [11,12] and APC/C^Cdh1 ^inhibition [13]. However, the regulation of Cdc5 itself remains relatively uncharacterized. In response to DNA damage, Cdc5 is inhibited by Rad53 mediated phosphorylation at an unidentified site [14,15]. Phosphorylation of T242 by Cdk1 has been reported to be essential for viability and mitotic activity [16], though this is disputed [1]. Nine other sites have been reported to be phosphorylated during mitosis [16], of which one, T29, when mutated to alanine stabilizes Cdc5 during G1 [17]. Four of these sites are clustered within the first 70 amino acids of Cdc5, suggesting modification of this region may be of importance to the regulation of Cdc5.

### Cdc5 stability is regulated by phosphorylation of T23

**Figure 1:**
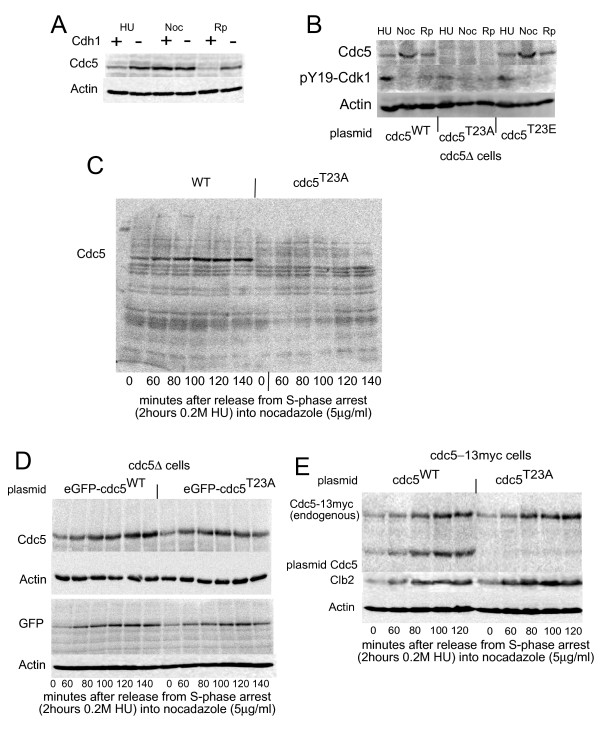
**A: The T23A mutation destabilizes Cdc5**. Wild-type and *cdh1Δ *cells were arrested in S-phase (0.2 M hydroxyurea), metaphase (5 μg/ml nocodazole) or early G1 (1 μg/ml rapamycin) for 2.5h and probed with anti-Cdc5 (Santa Cruz y300). **B: ***Cdc5Δ *cells [3] expressing Cdc5 from the indicated plasmids [16] were arrested in S-phase (0.2 M hydroxyurea), metaphase (5 μg/ml nocodazole) or early G1 (1 μg/ml rapamycin) for 2.5h. **C: **A full blot was probed with anti-Cdc5 to check for the possibility of proteolytic processing of *cdc5^T23A^*. Cells were released from S-phase and rearrested at metaphase. **D: ***Cdc5*Δ cells expressing GFP-Cdc5^WT ^[3] or GFP-*cdc5*^T23A ^were synchronized in S-phase, released and rearrested at metaphase. **E: **Cdc5-13myc cells [3] expressing Cdc5^WT ^or *cdc5*^T23A ^were synchronized in S-phase, released and rearrested at metaphase.

Cdc5 degradation by the APC/C^Cdh1 ^ubiquitin ligase is mediated by two D-boxes (RxxL) located within the N-terminal 70 amino acid region. Cdc5 levels oscillate during the cell cycle, from low in G1 and S-phase to elevated in G2/M [7,18]. Cdc5 levels in *cdh1Δ *cells are invariable throughout the cell cycle and comparable to levels of Cdc5 in wt metaphase cells. (Figure 1A). Phosphorylation on or adjacent to APC/C specific D-boxes could have a role in the regulation of substrate degradation. Cdc5 has several known phosphosites in its N-terminal part that could be involved in such a regulation. We have previously reported that a mutation of the T29 phosphosite dramatically stabilizes Cdc5 and completely eliminates its APC/C specific degradation [17]. In this report we studied the T23 phosphosite. Cells expressing *cdc5^T23A ^*[16] as the sole Cdc5, demonstrate normal nuclear division, septin, mitochondrial, and tubulin dynamics and undergo normal cytokinesis.

The abundance of wild type Cdc5 and *cdc5^T23A ^*protein during the cell cycle was examined by treating cells with hydroxyurea (S-phase arrest), nocodazole (metaphase arrest), or rapamycin (early G1 arrest). Curiously, *Cdc5^T23A ^*protein was undetectable under all these conditions **(**Figure 1B**)**. Examination of whole blots with anti-Cdc5 ruled out proteolytic processing **(**Figure 1C**)**. Moreover *cdc5^ΔN70^*, which lacks this phosphorylation site [12], migrates at the expected size (Rosella Visintin, personal communication). As Cdc5 is an essential protein we assume that *cdc5^T23A ^*cells still express it, albeit at an undetectable level.

The lack of detection of this essential protein could be due to two reasons - the first is that the antibody used does not bind to this mutant. We considered this unlikely, as the antibody used is a polyclonal antibody against the entire C-terminus of Cdc5. The other possibility is that the T23A mutation is, or has, introduced a degron. To test these ideas we expressed GFP-Cdc5^WT ^[3] or GFP-*cdc5*^T23A ^from the Cdc5 promoter either as the sole Cdc5 or in addition to an endogenous copy of Cdc5. Surprisingly GFP-*cdc5*^T23A ^was detectable, both by anti-GFP and by anti-Cdc5 **(**Figure 1D**) **and accumulated normally. This indicates that the T23A mutation indeed introduced a degron and that the degron was masked by the N-terminal fusion of the GFP protein.

To determine whether the instability of *cdc5^T23A ^*has an effect *in trans *- on other copies of Cdc5, *cdc5*^T23A ^was expressed in cells containing endogenous Cdc5^13myc^, which has reduced electrophoretic mobility. Accumulation of endogenous Cdc5^13myc^, and of Clb2, was unaffected by the presence of *cdc5*^T23A ^**(**Figure 1E**)**, suggesting that this mutation acts only in *cis*.

**Figure 2:**
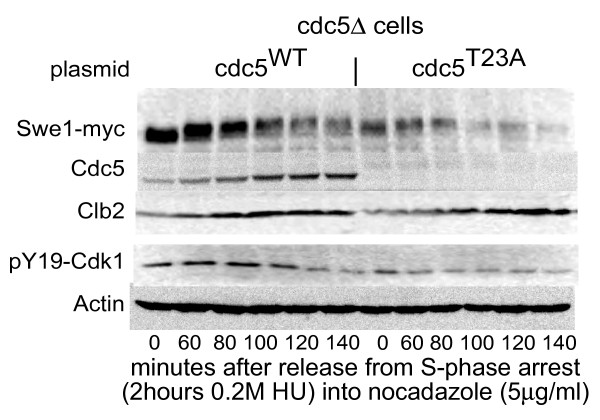
**Swe1 is degraded faster in *cdc5*^T23A ^cells**. *Cdc5Δ *cells expressing Cdc5^WT ^or *cdc5*^T23A ^were synchronized in S-phase, released and rearrested at metaphase. Rabbit anti-Clb2 was a kind gift from A. Amon. Swe1 was 6myc tagged by integration of pRS305 Swe1-6myc cut with SnaBI [26] and detected using 9E10 mouse anti-myc (a kind gift of M. Goldberg).

The degradation of Swe1, a kinase that phosphorylates Cdk1 on Y19 and inhibits its activity, requires Cdc5 [19]. In fact Swe1 degradation is one of the two essential functions of Cdc5 [3]. As *cdc5*^T23A ^protein levels were undetectable, we used Swe1 levels as a measure for Cdc5 activity. Cells were synchronized with hydroxyurea and released into nocodazole. Surprisingly, Swe1 levels were reduced in *cdc5*^T23A ^cells, even in hydroxyurea, and Swe1 was rapidly eliminated upon release from S-phase arrest (Figure 2A). Phosphorylation of Cdk1 on Y19 was likewise diminished, as could be expected from the reduced levels of Swe1. Swe1 degradation thus indicates that the *cdc5*^T23A ^protein must be present and active (and possibly even hyperactive) in cells. Accumulation of Clb2 was normal and, as we have already shown above, *cdc5^T23A ^*was not detected.

### Cdk1 is required for maintenance of Cdc5 levels in G2

**Figure 3:**
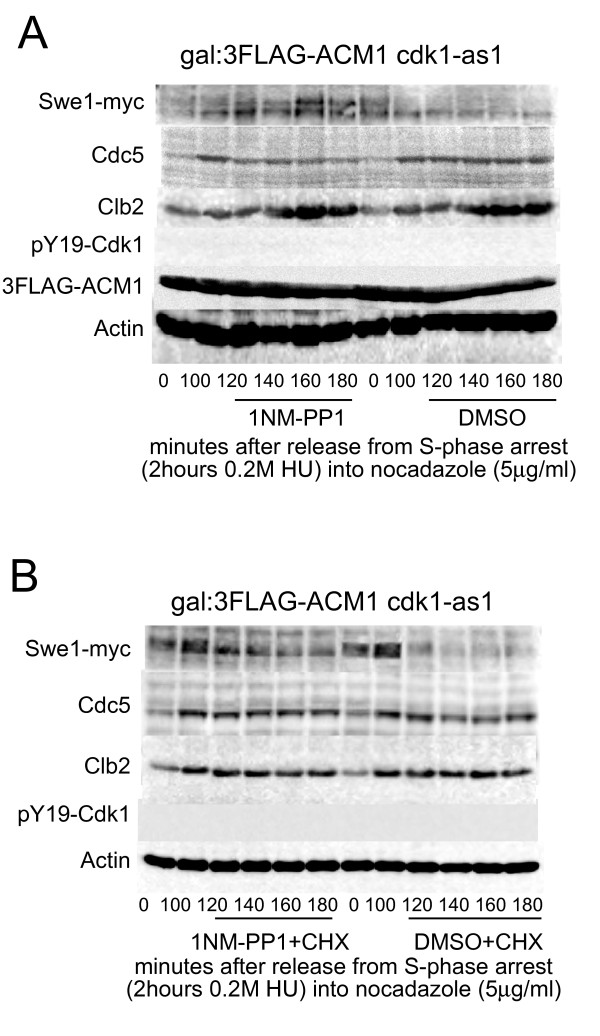
**A: Cdk1 is required for maintenance of Cdc5 levels in G2**. Cdk1^as1 ^[23] cells were synchronized in S-phase with 0.2 M hydroxyurea and released for 100 minutes into G2 (Cdc5 levels are near maximal) in media containing 5 μg/ml nocodazole. 0.5 μM 1 NM-PP1 (from a 1 mM stock in DMSO) or an equivalent volume of DMSO was added. **B: **As **A**, except that at 100 minutes 200 μg/ml cycloheximide (CHX) was also added to both samples. The activity of CHX is confirmed by the fact that levels of Clb2 do not continue to rise as happens in its absence (Figure 3A).

T23 is the only full consensus Cdk1 phosphorylation site of Cdc5 (S/T-P-x-K/R) and is phosphorylated during mitosis [16]. Although Cdk1-Clb2 activity promotes transcription of Cdc5, Clb2 and other CLB2 cluster genes [20-22], it has not yet been ascertained whether maintenance of Cdc5 protein levels during G2/M is also Cdk1 activity dependent. Cells carrying the 1NM-PP1 analogue-inhibitable *cdk1*^as1 ^[23] were synchronized in S-phase with hydroxyurea and released for 100 minutes into G2 to allow Cdc5 to reach near maximum levels before the addition of 0.5 μM 1NM-PP1. Cells additionally overexpressed the Cdh1 inhibitor Acm1 [24] to prevent possible APC/C^Cdh1 ^mediated destruction of Cdc5 [7,14]. Upon inhibition of Cdk1 activity, Cdc5 levels rapidly declined whereas levels of Clb2 remained stable **(**Figure 3A). Swe1 gradually increased in abundance in 1NM-PP1 treated cells. Therefore, Cdk1 activity seems to be required for maintenance of Cdc5 levels during G2, possibly by phosphorylation of Cdc5 on T23. The fact that cdk1 inhibition did not completely eliminate cdc5 levels as observed for the T23A mutation, can be due to the fact that cdc5 was initially phosphorylated and that this phosphorylation partially persisted for the duration of the experiment. It is also possible that other kinases can, to some extent, phosphorylate this site. Even if less likely it cannot be ruled out that the T23 residue is a structurally important residue whose mutations render general protein instability.

The decline in Cdc5 levels was prevented by addition of cycloheximide **(**Figure 3B), indicating that new proteins must be synthesized for the elimination of Cdc5 in the absence of Cdk1 activity. This Cdk1 inhibited, non-APC/C^Cdh1 ^destruction mechanism may also account for the residual instability seen in Cdc5 with both D-boxes mutated or in *cdh1Δ *cells [18], whilst Cdc5 with the first 70 amino acids deleted (*cdc5*ΔN70) is completely stable in G1 [7,12].

### Phosphorylation of T23 regulates Cdc5 accumulation

**Figure 4:**
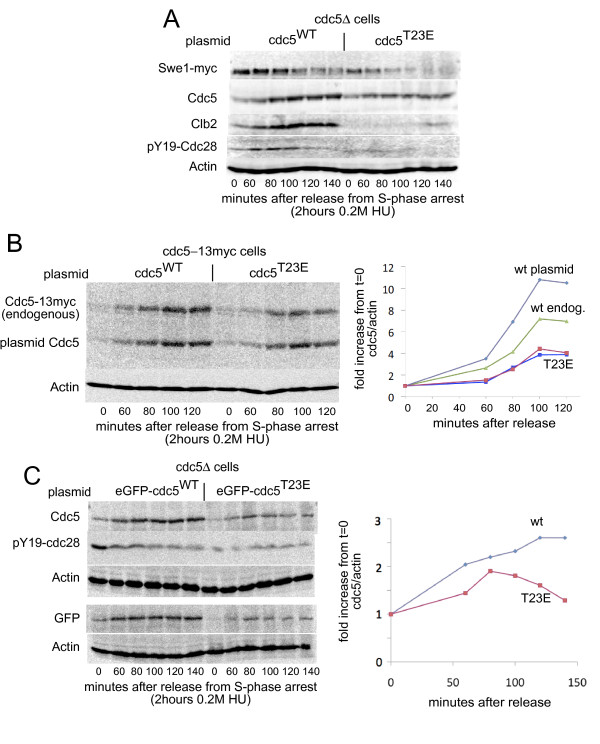
**Phosphorylation of T23 regulates Cdc5 accumulation**. **A: ***cdc5*Δ cells [3] expressing Cdc5^WT ^or *cdc5*^T23E ^[16] were synchronized in S-phase, released and rearrested at metaphase. **B: **Cdc5-13myc cells [3] expressing Cdc5^WT ^or *cdc5*^T23E ^[16] were synchronized in S-phase, released and rearrested at metaphase. **C: ***cdc5*Δ cells [3] expressing eGFP-Cdc5^WT ^or eGFP-*cdc5*^T23E ^were synchronized in S-phase, released and rearrested at metaphase.

We next examined the phospho-mimicking *cdc5*^T23E ^mutant [16]. *cdc5*^T23E ^cells (as their sole Cdc5) grew with normal morphology, separation, tubulin and septin dynamics (data not shown). S-phase arrested *cdc5*^T23E ^cells had reduced Swe1 levels and activity, and upon release Swe1 levels declined more rapidly than in wild-type cells, suggesting increased Cdc5 activity (see [1]). Interestingly, Clb2 was absent from these cells, and Cdc5 levels did not increase after 1 hour from release from S-phase arrest **(**Figure 4A**)**. In contrast to *cdc5^T23A^*, the presence of *cdc5^T23E ^*affects Cdc5 levels *in trans*, reducing Cdc5 accumulation by approximately 50% (Figure 4B). N-terminal tagging of *cdc5*^T23E ^with GFP did not suppress this reduced accumulation phenotype **(**Figure 4C**)**. Mutating a threonine (T) to glutamic acids (E) is generally considered a phospho-mimicking mutation. It is however hard to assess whether the effect of glutamic acid is like genuine threonine phosphorylation. In many cases such a mutation has no such effect, and behaves like a non-phosphorylatable alanine. The effect we observed here is clearly not like that of non-phosphorylatable alanine, as *cdc5*^T23E ^is considerably more stable than the undetectable *cdc5*^T23A^. However as *cdc5*^T23E ^is not as stable as wt cdc5 and leads to some other non-wt effects like clb2 disappearance, we might observe here an intermediate effect. It is also possible that phospho-mimicking of this site has one effect on stability and another on activation. The T23 site does not fall within the kinase domain of Cdc5 and its enhanced apparent activity is likely due to modified interactions with other proteins, rather than intrinsic changes in its kinase activity.

## Conclusions

APC/C^Cdh1 ^substrate proteins accumulate to maximal levels in metaphase and are either absent or present at much lower levels during the rest of the cell cycle. In *cdh1*Δ cells the level of these proteins is, in most cases, uniformly high throughout the cell cycle. Strikingly this level is comparable to their metaphase level in wild type cells. This observation suggests that some additional mechanisms are likely to cap the level of these proteins, in addition to APC/C^Cdh1 ^specific degradation. In this report, we have uncovered a potential mechanism that provides a negative feedback regulation of Cdc5 levels, a toxic protein when overexpressed [25], enabling cells to tolerate mild overexpression of Cdc5 (D. Morgan, personal communication). This mechanism is two-fold. Firstly, Cdc5 stability in G2 is Cdk1 kinase activity dependent. In the absence of Cdk1 kinase activity, Cdc5 is degraded by a novel, APC/C^Cdh1 ^independent mechanism that requires protein synthesis and which may account for the residual instability of D-box mutated Cdc5 [18]. Secondly, phosphorylation of T23 of Cdc5, presumably by Cdk1 [16], limits the rate and maximal accumulation of Cdc5, Clb2 and possibly other *CLB2 *cluster proteins. A potential mechanism could be via further phosphorylation of Ndd1/Fkh2 [2], mediated by *cdc5*^T23E ^to switch off *CLB2 *cluster transcription when a desirable level of *CLB2 *cluster proteins is reached.

## Competing interests

The authors declare that they have no competing interests.

## Authors' contributions

KJSL planned and performed the experiments and wrote the manuscript, MB wrote the manuscript and planned experiments. All authors read and approved the final manuscript.
